# Male immigration triggers increased growth in subordinate female meerkats

**DOI:** 10.1002/ece3.4801

**Published:** 2018-12-27

**Authors:** Constance Dubuc, Tim H. Clutton‐Brock

**Affiliations:** ^1^ Department of Zoology University of Cambridge Cambridge UK; ^2^ Department of Zoology and Entomology, Mammal Research Institute University of Pretoria Pretoria South Africa

**Keywords:** breeding competition, developmental flexibility, inbreeding avoidance, strategic growth

## Abstract

There is increasing evidence that some vertebrates can adjust their growth rate in relation to changes in the social context that affect their probability of breeding. Here, we show that, in meerkats (*Suricata suricatta*), which are singular cooperative breeders, subordinate females increase in body mass after their father is replaced as the dominant male in their natal group by an immigrant male, giving them regular access to an unfamiliar and unrelated mating partner, while their brothers showed no similar increase nor did subordinate females living in other stable groups (where male immigration did not occur did) in this time period. Moreover, subordinate females showed a greater increase in growth rate when their father was succeeded by an unfamiliar immigrant male than when he was replaced by a familiar male who was already resident. These results suggest that female meerkats can adjust their rate of growth to changes in the kinship composition of their groups that provide them with increased access to unrelated breeding partners, which may occur in other mammals as well when breeding opportunities change.

## 1 BACKGROUND

Recent research on social vertebrates shows that individuals of some species adjust their growth rate in relation to variation in the costs and benefits of body mass generated by changes in their social environment. In some group‐living fish, subordinates whose mass approaches that of rank neighbors immediately above them in social hierarchies reduce their growth rate to avoid becoming the target of persistent aggression (Ang & Manica, 2010; Buston, 2003; Heg, Bender, & Hamilton, 2004; Wong, Munday, Buston, & Jones, 2008). In some polygynous mammals, males increase growth rates leading to in overall mass and in the development of secondary sexual characters when they acquire their own breeding territory (Clutton‐Brock, 2016; Emery Thompson, Zhou, & Knott, 2012; Oliveira, Canário, & Ros, 2008; Setchell, 2008) or when their social status changes.

Where reproductive competition between females is unusually intense, strategic increases in growth also occur in females. For example, in naked mole‐rats (*Heterocephalus glaber*) and Damaraland mole‐rats (*Fukomys damarensis*), where regular breeding is restricted to one dominant female in each group and competition for the breeding role is intense, changes in the social status of females from non‐breeding subordinate to breeding dominants are associated with renewed growth and with changes in body mass, size, and shape (O'Riain, Jarvis, Alexander, Buffenstein, & Peeters, 2000; Young & Bennett, 2010). Similarly, in meerkats, females that acquire the breeding position in their group subsequently show a period of increased growth that usually establishes them as the heaviest member of their sex in the group (Clutton‐Brock et al., 2006; Huchard, English, Bell, Thavarajah, & Clutton‐Brock, 2016; Russell, Carlson, McIlrath, Jordan, & Clutton‐Brock, 2004). In meerkats (*Suricata suricatta*), subordinates of both sexes also respond to experimentally induced increases in the growth of individuals ranked immediately below them in social hierarchies by raising their own rate of growth and food intake (Huchard et al., 2016).

In species where a single breeding female and a single breeding male monopolize breeding in each group, the death or removal of the resident breeding male and the immigration of one or more unrelated males lead to changes in the hormonal status, behavior, and breeding activity of subordinate females, many of which are the daughters of the resident breeding male (Clutton‐Brock, 2016). For example, in groups of Damaraland mole‐rats, the experimental replacement of the resident breeding male by an unrelated immigrant leads to increases in reproductive hormones and sexual proceptivity in subordinate females as well as increased aggression (Cooney & Bennett, 2000; Jacobs, Reid, & Kuiper, 1998). Similarly, in meerkats, where subordinate females rarely breed with closely related or familiar males (Nielsen et al., 2012), the replacement of the resident breeding male by immigrant males from other groups leads to increases in sexual proceptivity in natal subordinate females (Carlson et al., 2004; Clutton‐Brock et al., 2001; O'Riain, Bennett, Brotherton, McIlrath, & Clutton‐Brock, 2000).

**Figure 1:**
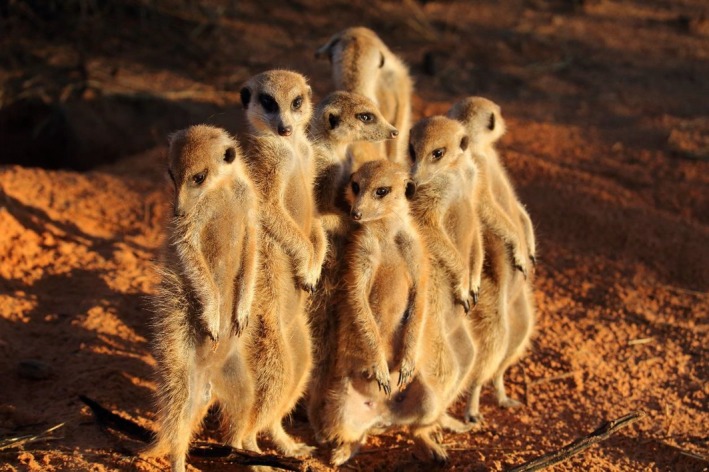
Family unit of cooperative breeding meerkats (*Suricata suricatta*) photographed at the Kalahari Meerkat Project (KMP) in the Kuruman River Reserve, Northern Cape, South Africa (photo credit: Constance Dubuc)

In this paper, we investigate whether the immigration of males to established breeding groups of wild Kalahari meerkats (Figure 1) is also associated with increases in the growth of resident subordinate females. Since relative body mass predicts the probability to breed as subordinates (Clutton‐Brock et al., 2001; Clutton‐Brock, Hodge, & Flower, 2008), we emit the hypothesis that subordinate female meerkats strategically adjust their rate of growth when at these times when opportunities to breed emerges in their natal group, like they do when they newly become member of the dominant pair (see Huchard et al., 2016).

Kalahari meerkats are cooperative breeders living in groups of 2–50 including an unrelated (or distantly related) breeding pair, which are dominant to all other group members of the same sex, and a variable number of closely related and highly familiar non‐breeding subordinates of both sexes that help to rear young born to the breeding pair (Clutton‐Brock & Manser, 2016). The breeding pair monopolize reproduction in their group almost entirely (Spong, Hodge, Young, & Clutton‐Brock, 2008) producing up to three litters of 2–7 pups per year. Dominant females can live for up to 12 years while the lifespans of dominant males are around 50% shorter than those of females (Clutton‐Brock et al., 2006). Subordinate females sometimes attempt to breed after mating with roving males from neighboring groups but rarely do so successfully (Clutton‐Brock & Manser, 2016; Nielsen et al., 2012). While subordinate individuals remain in their natal groups, they seldom breed with members of their natal group, to whom they are usually closely related (Clutton‐Brock & Manser, 2016; Nielsen et al., 2012). There are no cases of breeding with close relative, and in all reported cases where females bred with related males, they were already distant relative with no prior experience living in the same group together (Nielsen et al., 2012). Subordinate females are eventually evicted by the resident dominant female before they are 4‐years‐old and then either form a new breeding group or die as they are seldom able to join established breeding groups by resident females. Over three‐quarters of females that reach breeding age consequently fail to acquire dominant breeding positions at any stage in their lives (Clutton‐Brock & Manser, 2016). After the death of a dominant female, older subordinate females compete for dominance before the oldest and heaviest subordinate resident usually assumes dominance, and then remains and breeds in the group for the rest of her life (Clutton‐Brock & Manser, 2016; Duncan, Gaynor, & Clutton‐Brock, 2018). Subordinate males that have been born in the group very rarely mate or breed either with the dominant female or with subordinate females in their group of origin (Griffin et al. 2003; Spong et al, 2008). In contrast to subordinate females, subordinate males are seldom evicted from their natal groups and leave voluntarily between the ages of two and five and subsequently either join other breeding groups, form new groups with dispersing females or die. After dominant males die, they are usually replaced by one or more immigrant males though, in around a quarter of all cases, a resident natal male acquires the dominant position (Clutton‐Brock & Manser, 2016). These resident natal dominant males rarely breed in the group (Leclaire, Nielsen, Sharp, & Clutton‐Brock, 2013; Spong et al., 2008) and are usually replaced by an immigrant male within a year and, if dominant females breed in the meantime, it is usually with roving males from other groups that are not close relatives (Clutton‐Brock & Manser, 2016; Spong et al, 2008).

The arrival of unrelated and unfamiliar immigrant males is associated with increases in the probability that subordinate females will breed (Clutton‐Brock et al., 2001) and so might be expected to trigger increases in growth in resident subordinate females. Our analysis examines changes in the rate of growth in 183 subordinate females following the replacement of the resident breeding male in their group. To check that any increase in the growth of subordinate females following the immigration of unrelated males in their group was a consequence of increased opportunities to breed rather than of disturbance associated with the immigration of unfamiliar animals, we compare the growth of female subordinates (whose opportunities to breed are enhanced by the arrival of unrelated males) with that of male subordinates of the same age, in the same groups (whose opportunities to breed are unaffected or even reduced by the arrival of new males). In addition, we compare the growth of female subordinates in groups where male immigration occurred with that of female subordinates of similar ages in groups where resident males maintained their position and no male immigration occurred. Finally, we compare the growth of female subordinates in groups where a male immigrant replaced the resident dominant male with the growth of subordinate females in groups where the resident dominant male was replaced by a familiar male who was already resident.

Increases in growth and mass and changes in body shape associated with social conditions that provide subordinate females with breeding opportunities may generate reproductive benefits of two kinds. First, they may improve the capacity of females to gestate relatively large litters: for example, in Damaraland mole‐rats, increases in growth of individuals that acquire breeding positions are associated with the development of a more elongate body shape (Young & Bennett, 2010) and larger individuals conceive, gestate and produce larger litters (Thorley, Katlein, Goddard, Zöttl, & Clutton‐Brock, 2018). Alternatively, in species where the competitive ability of individuals is positively related to their body mass, they may increase their capacity to compete successfully with other group members of the same sex to establish and maintain themselves as dominants. For example, in meerkats, non‐breeding subordinate females respond to increases in the growth and mass of individuals immediately below them in size‐related hierarchies by increasing their own food intake and growth rate (Huchard et al., 2016). While the comparisons described above indicate whether any increases in the growth of subordinate females following male immigration are associated with increases in breeding opportunities, they do not allow us to tell whether increases in growth are a response to breeding opportunities per se or whether they are a response to increases in the probability of competition for the breeding role. Following the death of dominant females, subordinate females compete to replace her so that, if increases in female growth are a response to increases in the probability of competition for breeding opportunities, they should also occur after the death of dominant females. Conversely, if subordinate females increase their growth rates after male replacement by immigrants but not after female replacement, this suggests that any increases in growth probably serve to enhance the female's capacity to gestate and rear young. Consequently, we also compare the growth of resident subordinate females after the arrival of immigrant males with their growth after the death of resident dominant females.

## 2 METHODS

### 2.1 Study population

We used long‐term life history and mass data of recognizable individuals collected over 18 years by the Kalahari Meerkat Project (KMP) in the Kuruman River Reserve, Northern Cape, South Africa (Clutton‐Brock & Manser, 2016). Since 1997, the project has monitored 10–15 groups of meerkats per year. All groups are visited on 2–4 mornings per week, when all group members are identified, and life history data are collected (pregnancies, births, dominance changes, evictions, dispersal, deaths). Subjects are also weighed on emergence from their sleeping burrow before foraging start (morning mass), again after 3 hr of foraging (midday mass) and again before the animals entered their sleeping burrow. Ages of most individuals are known to within a week.

### 2.2 Parentage assignment

A 2‐ to 5‐mm tissue biopsy from the tail tip is taken from all individuals of the population when they first emerge from the natal burrow at approximately 3 weeks of age for genetic analysis (Spong et al., 2008). All sampled individuals are genotyped using up to eighteen variable microsatellite loci (Nielsen et al., 2012) and parentage is assessed using a combination of microsatellite data and phenotypic descriptors and two parentage inference programs: COLONY2 (Wang, 2004; Wang & Santure, 2009) and MASTERBAYES (Hadfield, Richardson, & Burke, 2006). Details of the molecular and pedigree construction and structure can be found elsewhere (Huchard et al., 2014; Nielsen et al., 2012). We consider parent‐offspring and full sibling dyads (*r* = 0.5) as closely related kin, while all other kin categories are considered as distantly related.

### 2.3 Samples

We limited our analyses to subordinate females for which both parents were confirmed by genetic analysis to be dominant to other group members of the same sex and lived to at least 12 months old, in order to exclude weaker individuals which may be unable to adjust growth rate. Using data collected between 1997 and 2015, we selected a total of 183 females, from 104 litters born in 19 groups from 28 different dominant females and 31 dominant males as potential subjects. In the groups from which this sample of animals was drawn, 15 dominant males were replaced by new dominant males in nine different groups, and 47 subordinate natal females and 55 subordinate natal males out of a total of 28 litters experienced the replacement of their fathers by another male when they were 6–36 months old (excluding subordinate females who were pregnant when their father was replaced as dominant male). Only cases where the mother remained dominant were included. All subordinate females who were present both before and after their father's death were included, including the beta female. Note that in all but one case, the dominant female (i.e., the mother of our subordinate female subject) became pregnant within the first month of tenure of the new dominant male, preventing us from examining whether their growth rate also increased when the dominant male was replaced. We considered as unfamiliar individuals who had never co‐resided in the same group in their lifetime.

Of these 47 females who experienced the replacement of their fathers by another male, a total of 18 experienced a new immigrant unfamiliar dominant male in their natal group—of these, six (from five litters in four different groups) experienced a new immigrant unfamiliar male immediately, while the remaining 12 (from eight litters in three different groups) first experienced a familiar, natal male as the new dominant male. The data set also contained 29 females (18 litters in seven groups) who only experienced a familiar dominant male after the death of their father. In all cases, familiar dominant males were related to the resident subordinate females, with 22.9% being closely related (offspring, full sib) and 77.1% being more distantly related to the dominant male (half‐brother, full uncle, half uncle). In contrast, there were no cases of closely related immigrant unfamiliar dominant males in our dataset: immigrant dominant males were unrelated to the subordinate females in 70.4% of cases and distantly related (full cousin, half‐nephew) in the remaining 29.6%. For some comparisons, we also selected as subjects the male littermates of the subordinate females that experienced new dominant males, including 55 subordinate males from 28 litters in eight groups.

From our initial dataset of 183 subordinate females, we also selected 41 subordinate females from 25 litters in six groups that had experienced the replacement of their mother as the dominant female (nine separate cases) while their father remained as the dominant male; females who became dominant were excluded from the analyses. In these cases, the new dominant female was always a resident and was thus a familiar female. Only cases where the father was still dominant were included.

Finally, we selected as controls 105 subordinate females from 59 litters which were resident in 14 stable natal groups (where their parents formed the breeding pair and were not replaced).

### 2.4 Growth of mass

To allow comparison across time periods and individuals in the analysis, we calculated monthly growth rates for all individuals in each month by dividing the difference between the average mass in grams (g) in the current and previous month of life by 30.5 days. Because growth of mass can be affected by the age of individuals as well as by current ecological conditions (English, Bateman, & Clutton‐Brock, 2012; English, Bateman, Mares, Ozgul, & Clutton‐Brock, 2014), we calculated their relative growth rate. To do this, we used all 183 subordinate females from the complete dataset described above and excluded data for months (a) when females were pregnant, (b) when females were absent from their natal group because they had been evicted (Dubuc et al., 2017), and (c) after females have competed for the dominant position (Huchard et al., 2016; Russell et al., 2004). We then computed the residuals to the regression line of Linear Mixed Models (LMM), setting monthly growth rate as the response variable, age and month of the breeding year as a continuous fixed effect and breeding year as a categorical fixed effect. We set the beginning of the year in October (month 1) because this is when the rainy season typically start. All three predicted variables had significant effects on monthly growth rate: age (*F*_1,3574_ = 222.117, *p* < 0.001), month of the breeding season (*F*_1,3574_ = 100.009, *p* < 0.001), and breeding year (*F*_1,3574_ = 7.486, *p* < 0.001). The relative growth rate of subordinate males was calculated separately using the same method. Positive values mean that individuals grow faster than other same‐sex subordinates of their age in the same time period, while negative values mean that they grow slower.

### 2.5 Social interactions

In contrast to primates and many other animals where average kinship between group members is low, aggressive interactions between adult meerkats are rare. Individuals guard food items and feeding holes that they are digging against other group members but “owners” usually win these interactions and social aggression in other contexts is usually restricted to brief periods when individuals compete for dominant breeding status. As a result, the frequency of aggressive interactions was too low for it to be possible to examine the effects of changes in group membership on the frequency of social aggression or on dominance relationships among subordinates.

### 2.6 Food intake

We estimated food intake of subordinate females using the difference in the body mass of individuals between dawn and midday morning sessions, divided by the time difference between the two measurements. Previous analyses have shown that this measure varies seasonally in relation to changes in rainfall and food availability. We then calculated average food intake in the last month of tenure of the father and in the first month of tenure of the new dominant individual. As food intake is also affected by age, rainfall, and ecological conditions, we also calculated the relative food intake of different individuals, using the same approach that we used to control for the effects of variation in age and ecological conditions in the growth rate (see above).

### 2.7 Statistical analyses

We used Linear Mixed Models to examine whether changes in the composition of the dominant breeding pair affected subordinates’ growth rate and food intake. In all models, growth rate was square‐root‐transformed to meet the model assumptions. When applicable, we used the post hoc pair‐wise comparison LSD test to locate the existing variation.

We compared the growth rate of subordinate females between three periods: the last month of tenure of their father, the first month of tenure of an immigrant dominant, and the first month of tenure of a resident familiar dominant male. We estimate growth over a relatively short period of time since previous observations showed that accelerated growth in subordinate meerkats may only last for a limited period (Huchard et al., 2016). We set “relative growth rate” as response variable, “dominant male category” as categorical fixed effect (father, resident or immigrant), and included as random effects “individual ID,” “litter name,” “mother ID,” “father ID,” “group name,” and “month” nested within “year.” The same statistical approach was used to compare the growth rate of subordinate females with their male littermates between these three periods and to examine whether the replacement of the mother was associated with a shift in growth rate in subordinate females: here “dominant female category” (fixed effect) was binomial (mother or familiar). We then compared the growth rate of subordinate females who experienced the replacement of their father as dominant male with the growth rate of other subordinate females living in stable groups of the population. We ran three models and compared growth rate between the two categories of females (a) in the last month of tenure of their father, (b) in the first month of tenure of a familiar male, and (c) in the first month of tenure of an immigrant male, setting “relative growth rate” as the response variable, “subordinate female category” as fixed effect (subject vs. control) and using the same random factors as described above. We also examined how long the predicted rise in growth rate lasted in subordinate females who experienced an immigrant dominant male by comparing “relative growth rate” (response variable) between four periods (categorical fixed effect): the last month of the father's tenure and the first, second, and third months of tenure of the immigrant new dominant male. The same random factors were used than in the previous models.

Finally, we examined whether the replacement of the dominant male was associated with variation in the food intake of subordinate females. To do this, we first examined whether “relative food intake” (response variable) varied between “dominant male categories” (categorical fixed effect; see above). Second, we then compared “relative food intake” (response variable) of subordinate females that experienced the replacement of their father in the first month of tenure of the new immigrant male with that other subordinate females of stable groups of the population (categorical fixed effect; subject vs. control). The same random effects as described above were used in these models. As opposed to the models examining growth (see above), no transformations were required.

Statistical analyses were computed with IBM SPSS Statistics 23. Alpha levels were set at 0.05 and analyses were two‐tailed.

## 3 RESULTS

**Figure 2:**
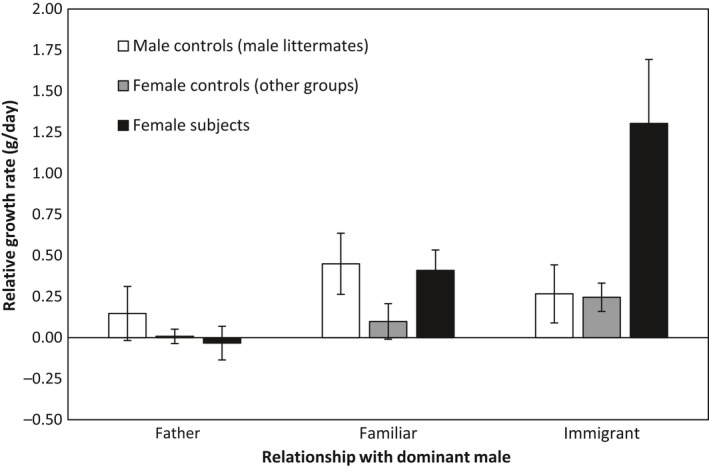
Differences in relative growth rate ±*SEM* (see text) in the month following the replacement of resident dominant males in (a) natal subordinate females, (b) their male litter mates, and (c) subordinate females living in other stable groups at the same time (female controls). The data presented here represent the relative growth rate in the last month of tenure of the father versus the growth rate in the first month of tenure of a familiar or an immigrant dominant male

Replacement of the father as dominant male in their natal group was associated with an increase in mass growth in subordinate natal females (*F*_2,92.796_ = 10.135, *p* < 0.001) (Figure 2). The magnitude of increases in the growth rates of subordinate females varied with the origin of new dominant males: there was an increase in the growth of subordinate females following the replacement of their father) by an immigrant unfamiliar male (0.350 ± 0.080, *df* = 97.679, *p* < 0.001) as well as following replacement of a resident dominant male by a familiar resident male (0.200 ± 0.083, *df* = 91.872, *p* = 0.018) (Figure 1). However, subordinate females grew significantly faster in the presence of a new immigrant unfamiliar dominant male than following replacement by a familiar natal male (−0.200 ± 0.083, *df* = 91.872, *p* = 0.018) (Figure 2). In the first month of tenure of an immigrant unfamiliar male, subordinate females grew 5.4 times faster than in the last month of tenure of their father and 3.2 times faster than in the first month of the dominance tenure of a resident familiar male.

In contrast, replacement of the mother as the dominant female was not associated with any consistent changes in the growth rate of subordinate females (0.234 ± 0.232, *F*_1,62.305_ = 1.016, *p* = 0.317), and there was no similar increase in the growth rate of subordinate natal males in the same groups (*F*_2,74.011_ = 0.886, *p* = 0.417) (Figure 2). Moreover, subordinates females experiencing a new immigrant dominant male also grew 5.3 times faster than other subordinate females living in stable groups over the same period (0.258 ± 0.064, *F*_1,68.967_ = 16.439, *p* < 0.001), but no difference was detected when we compared their growth rate in the last month of tenure of their father with that of females in other groups (−0.041 ± 0.022, *F*_1,116.752_ = 3.569, *p* = 0.061) or the first month of tenure of a resident familiar male (0.025 ± 0.041, *F*_1,57.151_ = 0.374, *p* = 0.543) (Figure 2).

**Figure 3:**
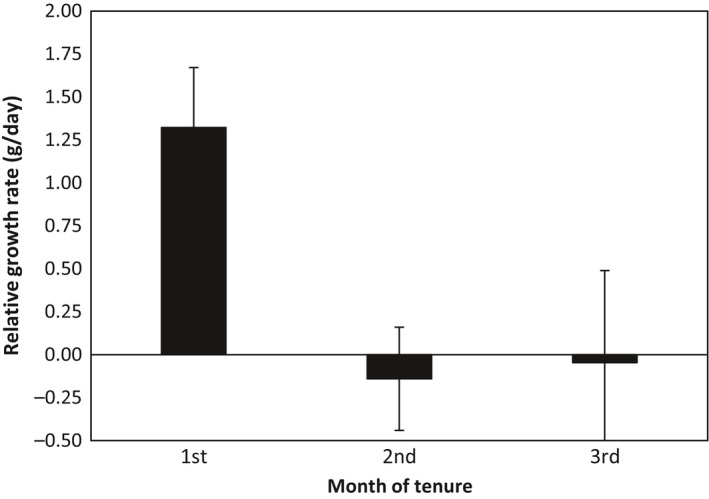
Average ±*SEM* relative growth rate of subordinate females in the first, second, and third month of tenure of a new immigrant male. In contrast to changes in body mass in month one, changes in body mass in months two and three were not significant

The rise in growth rate in subordinate females following the immigration of a new unfamiliar dominant male declined rapidly after the end of the first month of his tenure (*F*_3,44.895_ = 4.344, *p* = 0.009). In contrast to the first month of tenure, there was no difference in the growth rate of subordinate females in the last month of tenure of their father and their growth rate in the second (−0.070 ± 0.160, *df* = 45.134, *p* = 0.666) or third month (−0.191 ± 0.199, *df* = 45.110, *p* = 0.341) of tenure of the new immigrant unfamiliar dominant male (Figure 3).

Despite the increase in the growth rate of females after the replacement of their father, there was no significant increase in the daily food intake (as measured by the change in body mass between successively collected masses of the same individuals on the same day) of subordinate females (*F*_2,90.072_ = 0.645, *p* = 0.527). There was also no difference in food intake between subordinate females in the first month of tenure of an immigrant unfamiliar dominant male and that of other subordinate females of the population (−0.440 ± 0.898, *F*_1,60.620_ = 0.240, *p* = 0.626).

## 4 DISCUSSION

Our result show that, in Kalahari meerkats, resident subordinate females exhibit an increase in growth rate when their father is replaced by an immigrant unfamiliar male as dominant, while their brothers in the same groups and subordinate females living in other groups in the same population where individual males were not replaced showed no significant rise in growth over the same period. The stronger response of subordinate females to the presence of an immigrant male than to that of a natal dominant male also suggests that it is the presence of an unrelated male rather than the absence of their father that stimulates increases in growth. These results are consistent with the suggestion that increases in growth in subordinate females following the replacement of a resident dominant male represents a response to new breeding opportunities and other possible explanations of increases in the growth and mass of subordinate females appear unlikely. For example, it is unlikely that these increases are a consequence of conception and gestation in subordinate females since pregnant females show little increase in body mass during the first month of gestation (Sharp, English, & Clutton‐Brock, 2013) and as growth rate following the replacement of resident males declines in subsequent months (see Figure 3). In addition, it seems unlikely that the response of subordinate females to male immigration is a consequence of increased social instability since subordinate natal males, who are more likely than subordinate females to be the target of aggression from immigrant males show no similar increase in growth.

Like increases in growth following dominance acquisition (Huchard et al., 2016; Russell et al., 2004), increases in growth after the replacement of dominant males were not associated with increases in food intake, suggesting that increased growth may be associated with a change in the way that acquired energy is used, rather than by any modification of the amount of food obtained. Individuals may, for example, modify their behavior so that the costs of investment in cooperative activities are reduced (but see Huchard et al., 2016) or may increase their allocation of resources to muscle or skeletal mass relative to fat. More detailed analyses of the physiological, morphological and behavioral changes experienced by females in this period are needed to tease these different possibilities apart.

Evidence that subordinate females are able to increase their growth without increasing food intake suggests that they may normally grow at a slower pace than they are able to achieve, increasing growth only when breeding opportunities arise. There may also be ecological constraints on rapid growth in arid habitats characterized by low and unpredictable patterns of rainfall and high variation in food availability, where many cooperative breeders occur (Lukas & Clutton‐Brock, 2017). In these areas, subordinate females might gain by investing acquired energy into fat reserves rather than into muscle or bones in order to increase their probability to survive the next drought. Alternatively, increases in rates of growth may have deferred costs to rates of aging and survival (Metcalfe & Monaghan, 2001, 2003) or may lead to increased rates of aggression from other group members (Ang & Manica, 2010; Buston, 2003; Wong, Buston, Munday, & Jones, 2007; Wong et al., 2008).

The duration of periods of accelerated growth following the replacement of dominant males was similar to that of subordinate females experimentally challenged by the growth of a same‐sex littermate (Huchard et al., 2016), though it was shorter than that exhibited by newly dominant individuals, which can last for several months (Huchard et al., 2016; Russell et al., 2004). This could indicate that subordinate individuals are more closely limited than dominants in the period for which they can maintain accelerated growth. An alternative possibility is that investment in rapid growth may be proportional to the actual increase in the probability of breeding: since subordinate females exposed to an immigrant male still experience a lower probability of breeding successfully than dominant females, the investment in growth may not be increased to the same extent.

Our results show that subordinate females did not exhibit similar changes in growth rate when their mother was replaced as the dominant female. Since subordinate females commonly compete for the dominant position after the death of a dominant female and their body mass exerts an important influence on their success (Duncan et al., 2018), this suggests that the increase in growth is more likely to represent an adaptive adjustment to the probability of breeding and the demands of gestation than to increases in reproductive competition. In Damaraland mole‐rats, where females that acquire dominance show an immediate increase in growth the size of females is positively related to the size of their litters (Thorley et al., 2018). However, an alternative explanation could be that following the death of a dominant female, the oldest and heaviest subordinate female assumes dominance so quickly that there is no lasting stimulus for increased growth in females (see Duncan et al., 2018), whereas the immigration of an unrelated male provides subordinates with increased breeding opportunities over a much longer period (Clutton‐Brock et al., 2001; Spong et al., 2008).

Our results reinforce previous evidence of the effects of changes in social conditions on growth in social mammals (Huchard et al., 2016). Effects of social conditions on growth have been documented in a number of fish where growth commonly persists throughout the lifespan (Ang & Manica, 2010; Buston, 2003; Wong et al., 2008) but are uncommon in mammals which commonly show relatively little growth after individuals reach adulthood (Bennett, Jarvis, Aguilar, & McDaid, 1991; Karkach, 2006; Zullinger, Ricklefs, Redford, & Mace, 1984). However, a number of recent studies now show that the transition from non‐breeding to breeding status is associated with a renewal of growth in either or both sexes (Emery Thompson et al., 2012; O'Riain, Jarvis, et al., 2000; Young & Bennett, 2010) and these effects may be less rare than has been supposed. If so, they offer unusual opportunities for research into the mechanisms that control the extent and duration of growth.

## CONFLICT OF INTEREST

The authors declare no competing interests.

## AUTHORS’ CONTRIBUTION

C.D. and T.H.C.‐B. planned the study and wrote the paper; C.D. performed the analyses. Both authors approved the final version and are accountable for the work.

## Data Availability

All data used in our analyses will be archived on Data Dryad.
